# Drug Repurposing for Inclusion of COVID-19-Related Indication: Field Study of the European Medicines Agency’s Response to the Pandemic

**DOI:** 10.3390/pharmacy13060179

**Published:** 2025-12-10

**Authors:** Antonio Ivanov, Violeta Getova-Kolarova, Ines Hababa-Ivanova

**Affiliations:** Department of Organization and Economics of Pharmacy, Faculty of Pharmacy, Medical University-Sofia, 2 Dunav Str., 1000 Sofia, Bulgaria; v.getova@pharmfac.mu-sofia.bg (V.G.-K.); ines.hababa98@gmail.com (I.H.-I.)

**Keywords:** repurposing, COVID-19, EMA, crisis management, clinical trials, drug safety

## Abstract

As one of the biggest challenges for healthcare in the 21st century, COVID-19 placed a sustained and intense demand on the European Medicines Agency’s resources and required constant adaptation and mobilization of different regulatory processes. In this situation, drug repurposing appeared as a promising potential approach in quickly emerging health crises due to its main advantage of reducing the time and cost for addition of new indications since it uses products proven to be of high quality, safe, and effective. We performed an analysis of European Public Assessment Reports for medicinal products authorized for the SARS-CoV-2 infection by the European Medicines Agency, showing a total of eight products with this indication, three (37.5%) of which used repurposing as a mechanism for development (remdesivir, tocilizumab, and anakinra). The application of this mechanism by these medicines highlights the importance of the life cycle stage at which repositioning is undertaken, which resulted in different volumes of data submitted in the respective European Public Assessment Reports. The participation of organizations other than the marketing authorization holder in key stages in the drug development process of repurposed products was once again confirmed, which emphasizes the need to regulate this interaction.

## 1. Introduction

The infection caused by the SARS-CoV-2 virus, declared by the World Health Organization (WHO) as a COVID-19 pandemic on 11.03.2020, due to its increasing spread, is one of the biggest health crises of the 21st century. During the first years of the pandemic, the efforts and resources of the entire global healthcare system were directed toward discovering ways to influence and prevent the disease, thus granting authorization to a multitude of vaccines and medicines. This was further emphasized by factors such as unusually high economic incentives for treatment development, as well as accelerated and eased regulatory procedures for authorization. Since the first effective treatment would yield the highest economic benefits, the time pressure factor was exceptional for many of the stakeholders [1]. Mackenzie et al. further suggest that severe and readily transmissible new diseases can be successfully contained only with a multifaceted, multilateral, and multidisciplinary response, including the WHO, different networks of health-related organizations, national health services, academic institutions, technical institutions, and individuals [2]. Infectious disease outbreaks have historically led to widespread disruptions in routine essential health services. Disruptions due to COVID-19 responses led to excess deaths, including among different patient groups, primarily pediatric and geriatric. This necessitates maintaining up-to-date response and preparedness plans for health crises, the main element of which is the availability of appropriate medicinal products [3].

Repurposing (or repositioning) was defined for the first time by Ashburn and Thor as the mechanism for detection of new uses of authorized medicinal products or investigational products outside their original indication/s [4]. Drug repurposing (repositioning) is grounded in two major scientific paradigms: first, the post-human genome discovery that certain diseases share common biological mechanisms, and second, the concept of pleiotropic drugs. Advances in biomedical science now allow diseases to be characterized by their molecular profiles (e.g., genes, biomarkers, signaling pathways, environmental factors, and others), thereby enabling qualitative and quantitative assessment of similarities across distinct pathological conditions. In parallel, numerous medicinal products with long-standing clinical use are phenotypically well characterized. Their wide spectrum of effects stems from the pleiotropic nature of drug molecules, which interact with multiple biological targets, both primary and secondary. This property underlies the potential for a single medicinal product to be effective against multiple diseases, when a secondary biological target of the drug is implicated in the pathophysiology of another condition. Similar to diseases, drug molecules themselves can be systematically analyzed for structural or functional similarity, providing a rationale for initiating the drug repositioning process [5]. The history of drug repositioning began with serendipitous observations of unexpected effects during the use of a given medicinal product. Technological advances and the emergence of large-scale analytical methods have since provided substantial momentum to this approach. Today, the pharmaceutical industry adopts a far more rational and systematic strategy in its repositioning initiatives [6]. According to receptor theory, the interaction of a drug molecule with one or more targets elicits several biological effects that may be therapeutically beneficial for a given indication or may produce undesirable side effects. In disease-oriented drug repositioning, the therapeutic use of a drug is expanded from its original indication to a closely related indication. In target-oriented repurposing, the identification of a new indication is linked to a well-established therapeutic target. In drug-oriented repurposing, in contrast, a new target is identified, enabling the association of the drug with a novel therapeutic indication [7]. At the core of drug repositioning lies a systematic understanding of molecule–target interactions. Experimental identification of molecule–target interaction properties is limited by low efficiency in both scope and throughput, which has driven the development of in silico methods. Leveraging computational power, these approaches enable the detection of the most potent molecule–target interactions [8]. Apart from some inherent drawbacks of the drug repurposing, such as lower affinity of the target–molecule interaction due to molecular optimization and using data with lower accuracy and consistency, this approach has many advantages. Drug repositioning offers significant advantages in reducing the risk of failure, as a repurposed medicinal product has already demonstrated safety in preclinical models and in humans. Second, repurposing shortens the overall development timeline, since preclinical studies, safety assessments, and, in some cases, formulation development can be bypassed, having been previously completed. Widely cited estimates indicate that de novo drug development typically requires 10–17 years, whereas repositioning strategies may reduce this to 3–12 years. Lower investment requirements represent another major advantage, although costs vary depending on the developmental stage at which repurposing is initiated. While expenditures associated with phase III clinical trials and regulatory approval may not decrease substantially, costs for phase I and phase II studies are reduced to a far greater extent. Collectively, these factors allow for a faster and lower-risk return on investment. It has been estimated that the average cost of bringing a repurposed drug to market is approximately USD 300 million, compared with USD 2–3 billion for the development and authorization of an entirely new molecular entity [9,10]. Drug repurposing also offers substantial advantages in the treatment of rare diseases, a field in which unmet medical needs are often profound. Rare diseases represent a therapeutic area associated with high development risk; however, the use of already characterized medicinal products reduces development time, cost, and overall risk compared with the traditional de novo approach. In addition, clinical trials in rare diseases have distinct challenges, including the need for sufficiently large study populations to ensure adequate statistical power. This requirement is difficult to meet both because of the inherently low prevalence of these conditions and due to challenges in patient recruitment stemming from strict inclusion and exclusion criteria. Repositioning strategies can mitigate these limitations by enabling the incorporation of data from previous clinical studies conducted with the same molecule, thereby increasing the cumulative statistical power to levels necessary for regulatory approval [11]. Public health emergencies represent another area in which drug repositioning holds distinct advantages, particularly by markedly shortening development timelines. During outbreaks such as the 2014 Ebola epidemic in West Africa or global pandemics such as that caused by SARS-CoV-2, traditional drug development pathways are not the preferred approach, as they require prolonged periods of evaluation. In contrast, the use of previously studied medicinal products offers the possibility of providing an initial therapeutic response aimed at reducing overall public health risk. This approach enables the reallocation of resources toward the discovery of sufficiently effective, safe, and disease-specific therapies for treating or alleviating the symptoms of the epidemic or pandemic condition [12,13].

The European Medicines Agency (EMA), as part of the European Medicines Regulatory Network (EMRN), has established itself as a key player in the process of authorizing pandemic vaccines and therapeutic agents and monitoring their safety. The EMA also has a key role in crisis coordination activities, management of medicines shortages, provision of information on COVID-19 medicines, and generation of robust real-world evidence (RWE). The uninterrupted work of the agency was made possible thanks to the development of a crisis management plan in 2006 and its adjustments after health crises like the 2009 H1N1 influenza pandemic [14]. Despite this, as every health crisis has its specifics, the COVID-19 pandemic posed difficulties, like the need for constant adaptation to emerging scientific data, remote working, communicating uncertainty in real time, and counteracting misinformation by providing authoritative reference data and reports. For example, one of the Food and Drug Administration’s (FDA) innovations during the pandemic was to issue a number of temporary policies to support digital health innovation during the pandemic, such as guidance documents to expand the use of digital therapeutics for psychiatric disorders and medical devices for remote patient monitoring [15]. In addition, harmonization between medicine regulators remains an important feature to be pursued during a health crisis. From one perspective, this will ensure pharmaceutical companies submit the same data packages to the different authorities without the need for additional research. From another perspective, patients will obtain equal access to therapies. Ghadanian and Schafheutle confirmed that the EMA and FDA utilized regulations effectively but definitively showed the differences in timing between drug authorizations in these agencies during the COVID-19 pandemic [16]. In this turbulent period, waiting for a full data package in marketing authorization submissions would have significantly delayed access to much-needed medicines. Moreover, a significant reduction in deaths and hospitalizations was observed after the authorization of medicines and vaccines against the SARS-CoV-2 infection. There are many lessons learned and some of them refer to the methods used for collecting data for drug approvals. This includes the drawbacks from the fragmented nature of clinical trials, which are often small, underpowered, or with suboptimal design. The need for large, well-designed trials, including platform trials, that can provide the robust data needed to support decision making is clear. Randomized controlled trials remain essential for clinical development of new vaccines or therapies, also in a pandemic context, but they can benefit from complementary evidence from clinical practice through the analysis of RWE [17]. Additionally, the scientific community unites around the need for enhanced interaction and alignment among stakeholders, the development of more pragmatic procedures, and bypassing the inherent limitations of a centralized trial infrastructure, which can all have a positive effect on access to medicinal products [18,19].

By conducting this research and analysis, we aim to study the effects of repurposing in drug development during the COVID-19 pandemic, synthesize the advantages and disadvantages, and identify external factors that may influence this method in the setting of a health crisis. In addition, our objective is also to confirm the impact of organizations other than the MAH (like academia, nonprofit organizations, etc.) in the process and the need for clear regulation for more effective collaboration.

## 2. Materials and Methods

We carried out research on the European Public Assessment Reports (EPARs) for authorized medicinal products available in the COVID-19 section on the European Medicines Agency website in Jul-2024. The methodology of the conducted analysis did not include an analysis of vaccines. Their repositioning is largely based on heterologous and non-specific effects for a general improvement of the body’s defenses, the precise mechanism of which is not fully discovered. In addition, the basic concept of these therapeutic agents is active immunization for prevention, which necessitates hyperactive specificity to the disease-causing agent and a reduction in the potential for repositioning.

The first EPARs to add an indication related to COVID-19 were used for the analysis, and specific sections were selected as a study subject:

- For evaluation of the quality data: “Discussion on chemical, pharmaceutical and biological aspects”, “Conclusions on the chemical, pharmaceutical and biological aspects”, and “Recommendation for future quality development”;
- For evaluation of the preclinical phase of drug development: “Non-clinical aspects”;
- For evaluation of the clinical phase of drug development: “Clinical aspects”;
- For evaluation of the safety data: “Risk Management Plan”.

The comparative part of the analysis was focused primarily on the qualitative and quantitative aspects of the different sections, where the quantitative comparison included evaluation of the absolute and mean values of different parameters. Where the “Clinical aspects” section did not provide enough data for proper comparison, we used only the clinical trial name/identifier and researched public databases for publications related to the trial or clinicaltrials.gov records.

We compared Kineret^®^ (anakinra) and the rest of the authorized medicinal products since it is a classic example of the use of repositioning due to its history of use of over 22 years and being authorized in 6 different therapeutic indications. In addition, we included RoActemra^®^ (tocilizumab) and Veklury^®^ (remdesivir) as a second line in the analysis since they are also authorized for use in patients with COVID-19 infection using repurposing.

## 3. Results

At the time of this study, eight medicinal products were authorized in the EU with COVID-19 as an indication, shown chronologically as per the date of issue of the marketing authorization (MA) in Table 1.

Extremely rapid development of the first drug (remdesivir) can be observed when comparing the dates for granting MA. It received a conditional MA only 115 days after the WHO declared the pandemic. Casirivimab/imdevimab, regdanvimab, tocilizumab, anakinra, and sotrovimab were next to receive MAs. The difference between the dates of issue for each of these five medicinal products can be considered relatively negligible compared with the number of days since the COVID-19 pandemic was announced. In this group, two of the drugs also used repositioning as the drug development approach, demonstrating its importance in addressing health crises. Paxlovid (PF-07321332/ritonavir) is a combination of drug molecules (nirmatrelvir/ritonavir) and was the second antiviral product for treatment of COVID-19 to be granted a marketing authorization in the EU. A comparison between remdesivir and PF-07321332/ritonavir showed a difference of 574 and 200 days for receiving conditional MA and complete MA, respectively, highlighting remdesivir as the first antiviral molecule ready for use for treatment of SARS-CoV-2 infection. It is worth noting that this was observed amid the use of ritonavir as part of the PF-07321332/ritonavir combination, which has a history of established use, but the use of the newly developed molecule PF-07321332 (nirmatrelvir) requires a robust demonstration of efficacy and safety.

The authorization of medicinal products by regulatory agencies aims to maintain high levels of quality, efficacy, and safety standards. Examination of the quality summary sections of the assessment report also showed a distinction between repositioned and innovative therapies. The first important observation was the complete absence of submitted information on the quality of the repositioned products, anakinra and tocilizumab, both of which have an established use in the EU. This fact allows the Committee for Medicinal Products for Human Use (CHMP) to consider these parts of the dossier acceptable without requiring additional data beyond those currently available. The assessment report for remdesivir showed a total of 11 issues identified in quality documentation that require specific obligations during remdesivir manufacturing. In addition, three recommendations for future quality development were also described. This is amidst the fact that remdesivir had not been authorized for use in the EU prior to the COVID-19 pandemic, but it has a history of development in similar therapeutic indications and, therefore, can be categorized as repurposed. The information on quality requirements in EPARs for the remaining medicinal products is summarized in Table 2.

In regard to non-clinical aspects, the medicinal products approved for use in SARS-CoV-2 virus infection showed similar results to the quality section analysis. In anakinra and tocilizumab, there was, again, an absence of new data submissions in the analyzed section of the evaluation reports, which is considered acceptable by the EMA due to long-standing use of the medicines. The remaining drugs, including remdesivir, provide in-depth preclinical programs that investigate multiple aspects such as ADME (absorption, distribution, metabolism, and excretion) characterization and different types of toxicity in a wide range of cell models and animal species.

**Figure 1:**
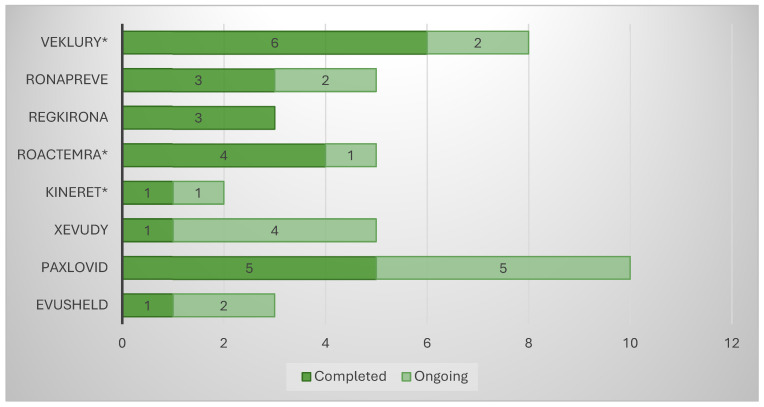
Clinical trials included in EPAR (* Medicinal product subject to repurposing).

**Figure 2:**
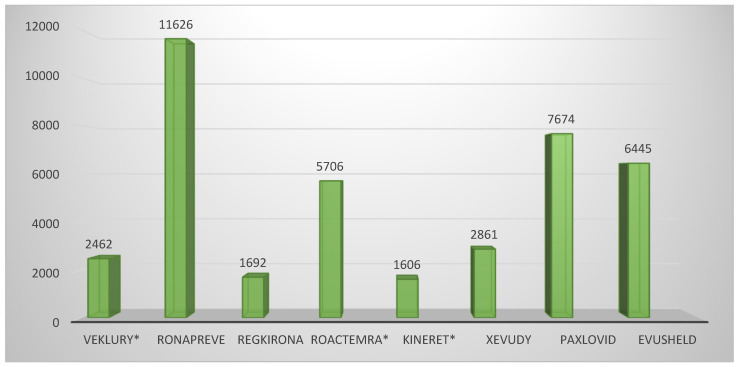
Total number of clinical trial subjects included in EPAR (* Medicinal product subject to repurposing).

**Figure 3:**
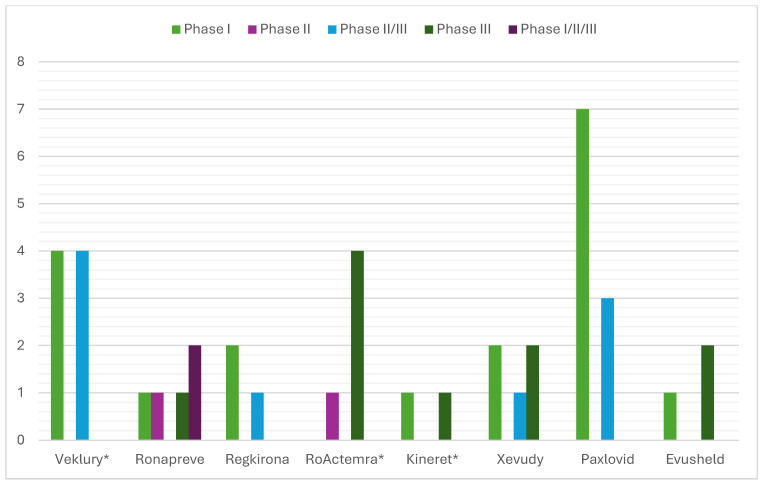
Distribution of clinical trials included in the EPAR focused on the phase (* Medicinal product subject to repurposing).

The analysis of the “Clinical aspects” section of the scientific discussion showed that the drug product with the lowest number of conducted clinical trials (CTs) was anakinra (two), where a COVID-19-related indication was added through repurposing (Figure 1). The other product using this approach, tocilizumab, showed five conducted CTs, on par with casirivimab/imdevimab and sotrovimab. Unlike the latter, however, the repositioned product submitted dominantly completed CTs (4/5 or 80%), while the innovative drugs reported 3/5 or 60% and 1/5 or 20%, respectively. A total of eight CTs were included in the EPAR of the third repositioned representative, remdesivir. Although this is a large number, it should be noted that 4/8 or 50% of them were conducted in the period of 2015–2019 and were phase I CTs to assess the safety, tolerability, metabolism, excretion, and pharmacokinetics of remdesivir. For this reason, remdesivir is classified as a repositioned medicinal product, as these are already accumulated data. However, it was necessary to include it in the submission due to the absence of a marketing authorization at the time of issuing the EPAR. For the clinical aspects section, the total number of subjects in clinical trials was another important parameter, part of the EPAR analysis. Again, anakinra showed the smallest number of participants (1606) compared with the other repositioned products, remdesivir and tocilizumab, ranking third (2462) and fifth (5706), respectively, in terms of the number of subjects enrolled in the clinical program (Figure 2). Analysis of the clinical trials distribution by phases also provided evidence of the effect of repositioning. For innovative products, there was a dominance of initial phases, such as phase I (particularly pronounced for PF-07321332/ritonavir and regdanvimab), as these therapies have not been authorized for use and need to provide positive data to demonstrate tolerability (Figure 3). For the repositioned product tocilizumab, there was a dominance of phase III CTs. This demonstrates the effect of using a proven-safe product, which does not require conducting additional phase 1 safety and tolerability trials. For anakinra, only one phase I trial was conducted to collect data in the new indication, and this result was consistent with tixagevimab/cilgavimab and casirivimab/imdevimab. In addition, although four phase I CTs for remdesivir were included in the EPAR, their aim was to demonstrate the safety and tolerability of the molecule in general rather than specifically in the indication associated with COVID-19.

**Figure 4:**
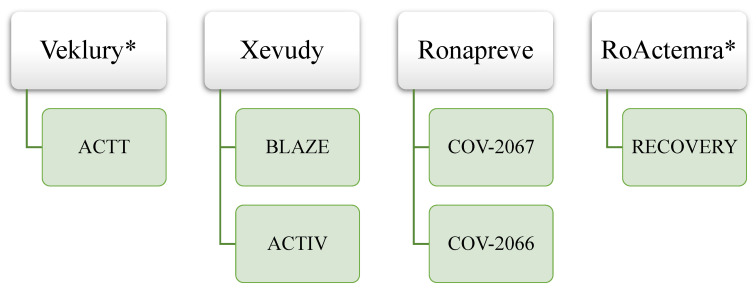
Clinical trials with platform/adaptive design or using master protocol included in EPAR (* Medicinal product subject to repurposing).

It is also interesting to note the impact of the COVID-19 pandemic on clinical trials with platform/adaptive designs or using a master protocol. Out of 41 total CTs included in EPARs, 6 (15%) were conducted using such designs. Examples of clinical trials with platform/adaptive designs for authorized medicines with SARS-CoV-2 indication are presented in Figure 4.

**Figure 5:**
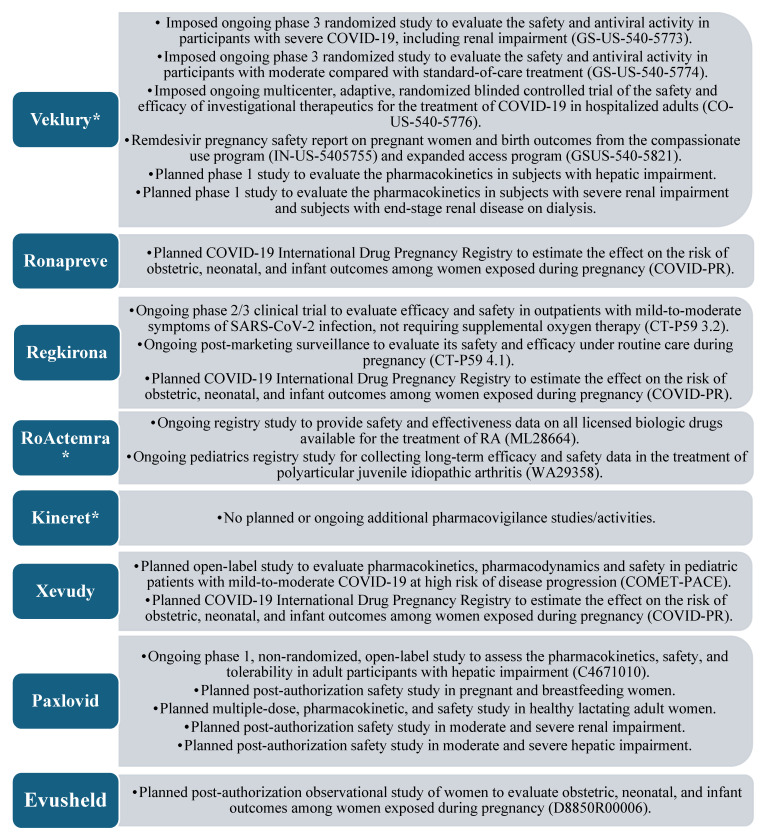
Summary of additional pharmacovigilance activities in EPAR’s pharmacovigilance plans of analyzed medicinal products (* Medicinal product subject to repurposing).

Safety is also part of the three most important features in the authorization of a medicinal product and is included in the EPAR, being described by a summary of safety concerns, risk minimization measures (RMMs), and the pharmacovigilance plan. For comparing the drug development and life cycle of repositioned and innovative medicines, the most comprehensive information is provided by the pharmacovigilance plan, as it describes ongoing and planned additional pharmacovigilance activities. The “Safety concerns” section can be considered specific to each drug molecule and cannot reflect the different drug development methods, whereas the “Risk minimization measures” section combines information from the previous two sections. Figure 5 presents a summary of the quantitative and qualitative results when comparing only the pharmacovigilance plan of all medicinal products.

## 4. Discussion

The analysis of EPARs showed that the authorized medicines for treatment of COVID-19 comprised one RNA polymerase inhibitor (remdesivir), one interleukin-1 receptor antagonist (anakinra), one combination of antivirals (PF-07321332/ritonavir), three monoclonal antibodies (regdanvimab, tocilizumab, and sotrovimab), and two combinations of monoclonal antibodies (casirivimab/imdevimab and tixagevimab/cilgavimab).

While this study did not show a dramatic difference in the time to obtain MA, a large impact of repositioning as a drug development mechanism in response to an emerging health crisis was observed. On the one hand, this was demonstrated by three out of eight medicines (37.5%) being authorized using this approach. On the other hand, the first authorized medicinal product to include a COVID-19-related indication was also a repositioning product and received an MA of 497 days before the next authorized ones. This occurred in the light of the EMA’s still-evolving regulatory initiatives for scientific advice for drug development programs that would subsequently favor the expeditious authorization of the remaining drugs. The absence of major gaps in the timing of granting MA can be explained by the global scale and strength of the health crisis, which demands directing enormous healthcare resources to drug development in this indication, as shown by von Delft and colleagues [20].

A major reason for the fast conditional MA issue for remdesivir was the use of drug repositioning in its development. Remdesivir (GS-5734) was developed by Gilead Sciences and emerged from a collaboration between Gilead, the U.S. Centers for Disease Control and Prevention (CDC), and the U.S. Army Medical Research Institute of Infectious Diseases (USAMRIID). They sought to identify therapeutic agents for treating RNA-based viruses that maintained global pandemic potential, such as those that, indeed, emerged following the initiation of the program, including EBOV and the Coronaviridae family viruses exemplified by Middle East respiratory syndrome (MERS) and severe acute respiratory syndrome (SARS). Although unapproved, the product is an antiviral therapy under development and proven to be safe in a number of preclinical studies and clinical trials [21]. Another major reason is the expedited regulatory procedures by the EMA and the use of so-called “rolling review”, where data for a drug product are submitted by the applicant and reviewed by the EMA “as it becomes available”. This has been shown by Marinus et al., who described the calling of extraordinary CHMP meetings and closer EMA–applicant communication as main advantages of rolling review assessment, which helps clarify the characteristics of the medicinal product [22].

The study on the quality section of the EPAR reinforced repositioning as a mechanism for rapid and accessible medicinal product development by using available data on product quality. Anakinra and tocilizumab demonstrated full use of already-collected quality data without providing data generated specifically for the new MA procedure. Meanwhile, remdesivir, PF-07321332/ritonavir, tixagevimab/cilgavimab, regdanvimab, casirivimab/imdevimab, and sotrovimab provided large data packages using time- and money-consuming tests. Farid and colleagues, in their study of costs in the biopharmaceutical industry, reported USD 40 million in expenses to develop and optimize the manufacturing, which is crucial for proof of quality [23]. Moreover, the additional financial resources required for post-approval quality improvement or addressing regulatory agency obligations/recommendations can also influence and raise the overall investment and the risk associated with it. The difference in the number of regulatory obligations/recommendations between representatives from the antibody MPs group and the antiviral group can be explained by the different parameters monitored in quality control. The antibodies are subjected to detailed characterization (physico-chemical, structural, immunological, and functional) during different stages of development, production, and storage by specific analytical methods, which can ensure batch-to-batch repeatability. In addition, antiviral substances are developed as solid dosage forms, which may require the inclusion of additional excipients to further stabilize and ensure proper delivery into the body.

Remdesivir, although using repositioning, also submitted a large volume of quality data due to performing “drug rescue” as a type of repositioning during the clinical development phase prior to MA issue. While overall remdesivir had a large number of quality obligations/recommendations (14 in total) compared with the antibody medicinal products (MPs) group, this number was lower than the other medicinal product in the antiviral group (PF-07321332/ritonavir), with a total of 22. The results of the summary sections of the quality in the EPAR also reinforce the advantages of repositioning. However, a correlation was found between the phase of the drug life cycle at which repositioning was undertaken and the amount of quality data submitted. The analysis showed that the EMA accepts the applications of authorized and repositioned products without the need for additional data, whereas repurposing an unauthorized product in its clinical phase can result in the need for a significant amount of data, close to that of innovative products.

A distinct advantage was again observed for the already authorized medicinal products, while no significant difference was found between the volume and complexity of the preclinical programs, e.g., remdesivir vs. innovative products. The latter observation may be explained by the fact that although data collection is available for a drug with still ongoing development, it is focused on a particular indication and may be difficult to adapt to a new emerging disease. Similar to quality requirements, the efficacy of repositioning in preclinical data is variable, with the main factor being the stage of the life cycle at which repositioning is undertaken. Other important factors may also include the accumulated knowledge of the new disease and the similarities between the indications for which the medicinal product is being developed and repositioned, respectively. Sultana and colleagues confirmed these factors as well, and their work further reinforces the need for good interaction between industry and academia in order to make precise use of limited resources during a health crisis [24].

In addition, by using a proven-safe drug molecule, repositioning allows conducting a smaller number of CTs directly targeting the desired indication, which was the case for remdesivir and its four phase II/III trials. Another important comparison is with PF-07321332/ritonavir, which had a larger clinical program, including two CTs more than remdesivir, and further reinforces the effect of repositioning. Thus, due to the use of a repositioned drug being developed for similar indications (SARS-CoV-1 and MERS-CoV), the drug regulator granted authorization with efficacy data in the indication of the new MA application without requirements for conducting additional phase 1 trials.

Comparison between the clinical programs of repurposed and innovative drugs also underlines the importance of academia and nonprofit organizations in repurposing. It is important to note that all clinical trials with anakinra were sponsored by an organization other than a pharmaceutical company (the Hellenic Institute for the Study of Sepsis), whereas the CT with the largest number of patients in remdesivir (RECOVERY) was sponsored by Oxford University. In contrast, the clinical program of innovative drugs is almost entirely sponsored by pharmaceutical companies. The only exception is the ACTIV-3-TICO trial for sotrovimab, which received public and private funding, with a design using a master protocol incorporating several subtests with different characteristics. This phenomenon was confirmed by Greenblatt et al., who showed that industry sponsorship played a limited role in supporting drug repurposing in the COVID-19 pandemic. Most of the sponsorship for clinical trials of repurposed drugs came from academic institutions, especially for drugs with generic competitors already on the market [25].

Regarding clinical trial design, the findings of our study are in line with those of Vanderbeek and colleagues, who confirmed the expansion in the use of complex and adaptive designs precisely during the pandemic. Their review showed a several-fold increase in adaptive clinical trials over the 2001–2019 period [26]. The authors described the ability to simultaneously compare a large number of therapies, rapidly add an investigational product, and even change the indication through a protocol amendment as key drivers of this phenomenon. In the largest clinical trial with the SARS-CoV-2 indication, the Randomised Evaluation of COVID-19 Therapy (RECOVERY) trial saw 11 approved drugs out of 14 participating [27]. Some of the benefits of conducting clinical trials with such designs match the goals of repositioning. Deloitte highlights, as pros, the reduction in risk to clinical programs through rapid testing of hypotheses and scientific questions and detecting early signals of failure without spending unnecessary resources. In addition, complex design reduces the time and cost of conducting the overall clinical program and mediates the accumulation of real-world data/evidence [28]. It is still noteworthy that platform/adaptive designs are still evolving and have challenges as well, like statistical considerations and the need for data-monitoring committees [29].

The comparative analysis of the clinical safety section of the EPAR strongly indicated the impact of repositioning as a method of drug development and the use of medicinal products that are proven to be safe. Anakinra showed a total absence of additional studies, part of the pharmacovigilance plan, and tocilizumab showed two non-interventional studies (NISs) of the registry type, which were intended to research drug safety information for use in diseases other than SARS-CoV-2 infection. These two observations provide evidence of the clear advantage of extending the application of a product with an established use, as there are usually no imposed additional drug safety-monitoring activities, nor activities specific to the new indication. Additional activities are listed in the EPARs of all other products, and in most cases, these represent NISs (registry type). It is also important to note that pregnancy use studies are planned or underway for all remaining drugs, once again demonstrating the small amount of information collected in the course of CTs for this population, also mentioned by Vousden and colleagues [30]. Thus, in an urgent health crisis, repositioning may emerge as the drug development strategy of choice in similar populations for which a sufficient amount of data is not traditionally collected in CTs.

Similar to the analysis of other parts of the evaluation reports, the results of the one on safety reinforce the importance of the life cycle phase of the medicinal product at which the repurposing is undertaken. Drug safety can be considered the most sensitive for this aspect of repositioning, which is evident from the large number of additional activities specifically in remdesivir, some of which are self-imposed by the regulator compared with the other products. In contrast with quality, where Veklury (remdesivir) has a large number of obligations/recommendations compared with the antibody MPs group, but less compared with PF-07321332/ritonavir from the antiviral group, remdesivir has the largest number of additional safety activities among all products.

Difference between drug classes (antiviral antibodies), as well as the indications for which they are approved (prevention or treatment of COVID-19), may lead to differences in clinical program data, quality, and safety in published European Public Assessment Reports and may be a possible limitation to our study. In addition, we reviewed only the first EPARs adding an indication related to SARS-CoV-2, with no follow-up of subsequent evaluation reports to update the information when administered in this indication.

## 5. Conclusions

The pandemic caused by SARS-CoV-2 virus demonstrated the impact of repositioning, with a total of 37.5% therapies authorized by the EMA based on such an approach. The data on several medicinal products clearly demonstrate the advantages of using a molecule proven to be qualitative, safe, and efficacious for development of new indications without the need for additional data and a large amount of clinical research. In addition, the importance of the life cycle stage of the drug product at which repositioning has been undertaken was also highlighted during this health crisis. Our study showed that a product that was already developed in the past but did not receive a marketing authorization may need to submit significantly larger quality, safety, and efficacy data package in the evaluation report compared with medicines with established use using repurposing. Nevertheless, a product that is repositioned prior to receiving authorization can become the first available to patients using regulatory mechanisms like scientific advice, rolling review, etc. With 50% of the medicines authorized for COVID-19-related indications included in clinical trials with a platform/adaptive design or using a master protocol, we confirmed the recent trend of their higher utilization. Finally, yet importantly, the involvement in drug development and clinical trials of organizations other than the MAH (like academia and nonprofit organizations) was represented clearly by funding the clinical trials for repurposed medicines, which, once again, confirms the need to regulate this interaction for more efficiency.

## Figures and Tables

**Table 1:** Chronological list of medicinal products receiving marketing authorization for indication related to SARS-CoV-2.

Medicinal Product	Date of MA Issue— Difference from Declaring COVID-19 Pandemic in Days	Repurposed Medicinal Product
Veklury * **(remdesivir)**	Conditional MA: 3 July 2020—115 MA: 8 August 2022—881	Yes
**Ronapreve (casirivimab/imdevimab)**	MA: 12 November 2021—612	No
**Regkirona (regdanvimab)**	MA: 12 November 2021—612	No
RoActemra * **(tocilizumab)**	MA: 7 December 2021—637	Yes
Kineret * **(anakinra)**	MA: 17 December 2021—647	Yes
**Xevudy** **(sotrovimab)**	MA: 17 December 2021—647	No
**Paxlovid** **(PF-07321332/ritonavir)**	Conditional MA: 28 January 2022—689 MA: 24 February 2023—1081	No
**Evusheld (tixagevimab/cilgavimab)**	MA: 25 March 2022—744	No

* Medicinal product subject to repurposing.

**Table 2:** Summary of the quality sections for non-repurposed medicinal products.

Medicinal Product	Issues Identified in Quality Documentation That Require Specific Obligations	Recommendations for Future Quality Development
**Ronapreve (casirivimab/imdevimab)**	-	3
**Regkirona (regdanvimab)**	-	1
**Xevudy** **(sotrovimab)**	-	5
**Paxlovid** **(PF-07321332/ritonavir)**	4	18
**Evusheld (tixagevimab/cilgavimab)**	-	13

## Data Availability

The original contributions presented in this study are included in the article. Further inquiries can be directed to the corresponding author.
